# Spectroscopic application of few-femtosecond deep-ultraviolet laser pulses from resonant dispersive wave emission in a hollow capillary fibre

**DOI:** 10.1039/d2sc02185d

**Published:** 2022-08-08

**Authors:** Nikoleta Kotsina, Christian Brahms, Sebastian L. Jackson, John C. Travers, Dave Townsend

**Affiliations:** Institute of Photonics & Quantum Sciences, Heriot-Watt University Edinburgh EH14 4AS UK d.townsend@hw.ac.uk; Institute of Chemical Sciences, Heriot-Watt University Edinburgh EH14 4AS UK

## Abstract

We exploit the phenomenon of resonant dispersive wave (RDW) emission in gas-filled hollow capillary fibres (HCFs) to realize time-resolved photoelectron imaging (TRPEI) measurements with an extremely short temporal resolution. By integrating the output end of an HCF directly into a vacuum chamber assembly we demonstrate two-colour deep ultraviolet (DUV)-infrared instrument response functions of just 10 and 11 fs at central pump wavelengths of 250 and 280 nm, respectively. This result represents an advance in the current state of the art for ultrafast photoelectron spectroscopy. We also present an initial TRPEI measurement investigating the excited-state photochemical dynamics operating in the *N*-methylpyrrolidine molecule. Given the substantial interest in generating extremely short and highly tuneable DUV pulses for many advanced spectroscopic applications, we anticipate our first demonstration will stimulate wider uptake of the novel RDW-based approach for studying ultrafast photochemistry – particularly given the relatively compact and straightforward nature of the HCF setup.

## Abstract 2

We exploit the phenomenon of resonant dispersive wave emission in gas-filled hollow capillary fibres to realize time-resolved photoelectron imaging measurements with an extremely short temporal resolution.

## Graphical abstract

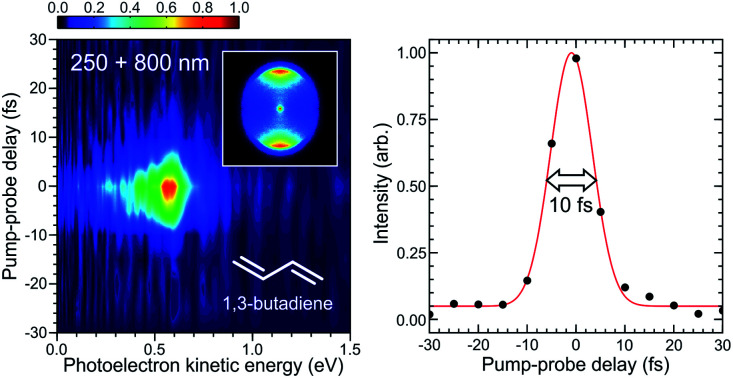

## Introduction

Our understanding of energy redistribution dynamics within the electronically excited states of molecules following ultraviolet absorption is underpinned by the Born–Oppenheimer approximation.^1^ This adiabatic model is based on the assumption that the vibrational and rotational motion of the molecular framework occurs on a much slower relative timescale to that of the electron motion – an effect that is a simple consequence of the significant difference in particle mass. The concept of electronic potential energy surfaces over which the nuclear motion evolves naturally stems from this starting picture, providing a mechanistic framework to discuss pathways along the reaction co-ordinates connecting initially prepared states to final photoproducts. The overall morphology of these potential energy landscapes then dictates the feasibility and rates of various possible outcomes. Non-radiative transitions between different potential energy surfaces along the reaction pathway may be facilitated by couplings between the nuclear and electronic degrees of freedom (*i.e.* a breakdown of the initial Born–Oppenheimer picture).^2–5^ These couplings, which may operate on timescales as short as a few femtoseconds, are critical drivers in many fundamentally important photophysical and photochemical processes. This leads to considerable interest in developing a more detailed understanding of such *non-adiabatic* behaviour. For example, the link to chemical structure and mechanistic function is relevant to numerous examples of biological, medical, environmental, and technological significance.^6–14^

Femtosecond laser systems provide an optical source with pulse durations comparable to the timescales of internal molecular motion. The evolution along the reaction co-ordinate may therefore be followed in real time through use of pump–probe techniques, and such approaches are now used routinely in the spectroscopic study of non-adiabatic molecular dynamics. Time-resolved photoelectron imaging (TRPEI) is a powerful variant of this general approach.^15–17^ Here photoelectrons ejected from a molecular sample are mapped onto a position-sensitive detector, generating a series of highly differential energy- and angle-resolved snapshots at incrementally varied pump–probe delay times. This yields information that is particularly instructive in revealing subtle mechanistic details of the dynamical pathways operating in a wide range of molecular species.^18–26^ Extremely short deep ultraviolet (DUV) laser pulses are an important tool for next-generation experiments in ultrafast spectroscopy which aim to develop an even deeper understanding of electronic and non-adiabatic dynamics in complex systems. To address relevant transitions in a wide variety of samples and with sufficient time resolution requires sources of wavelength-tuneable, few-femtosecond laser pulses across the deep and vacuum ultraviolet (DUV and VUV, 300–100 nm). While third-harmonic generation driven by few-cycle infrared (IR) pulses can generate DUV pulses as short as 2 fs,^27,28^ the conversion efficiency achieved with this approach is at most around 0.1%, and the wavelength is fixed by that of the fundamental driving pulse – a limitation which will become even more important as ultrafast experiments are increasingly driven by Yb-based laser systems at higher repetition rate and longer driving wavelength than hitherto predominant Ti:sapphire lasers. Alternatively, the use of (non-collinear) optical parametric amplifiers in conjunction with frequency doubling schemes can provide the required DUV tunability. This can achieve pulses durations of less than 10 fs, although requires careful management of the phase matching conditions in extremely thin birefringent crystals.^29,30^

The phenomenon of resonant dispersive wave (RDW) emission in gas-filled hollow-core waveguides is inherently wavelength-tuneable over the desired DUV and VUV region. First developed in hollow-core photonic crystal fibres (HC-PCFs),^31,32^ RDW emission in the DUV has more recently been scaled in energy by moving to hollow capillary fibres (HCFs) with much larger cores.^33^ HCFs are free of the resonances intrinsic to guidance in HC-PCFs and so the wide transparency window provided by atomic fill gasses allows for continuous tuneability from the VUV to the near IR^34^ with conversion efficiencies as high as 15%.^33^ Furthermore, atomic gasses are not susceptible to irreparable optical damage at high intensities, providing an attractive alternative to solid-state non-linear materials. Using all-optical pulse characterization, it has previously been demonstrated that RDW emission in HC-PCFs can generate nearly transform-limited 3 fs pulses when the waveguide is coupled directly to a vacuum system, creating a decreasing pressure gradient.^35^ Because the main difference between RDW emission in HCFs and HC-PCFs is one of scale, with similar underlying dynamics, it should be possible to obtain similarly short pulses in HCF; a prediction supported by numerical simulations.^33,36,37^ Although we have previously demonstrated the suitability of RDW-based sources for TRPEI applications in terms of tunability and stability,^38^ the significant potential advances in temporal resolution have not yet been exploited experimentally. In this work, we generate few-femtosecond DUV pulses *via* RDW emission in a vacuum-integrated HCF. We characterize the output pulses using a TRPEI cross-correlation measurement based on non-resonant two-colour DUV-IR multiphoton ionization of the 1,3-butadiene molecule. This demonstrates that RDW emission in HCFs can generate the extremely short pulses required for next-generation ultrafast spectroscopy experiments. To further reinforce this point we then present an initial TRPEI measurement investigating the excited-state dynamics operating in the *N*-methylpyrrolidine molecule following DUV absorption.

## Optical source

**Fig. 1:**
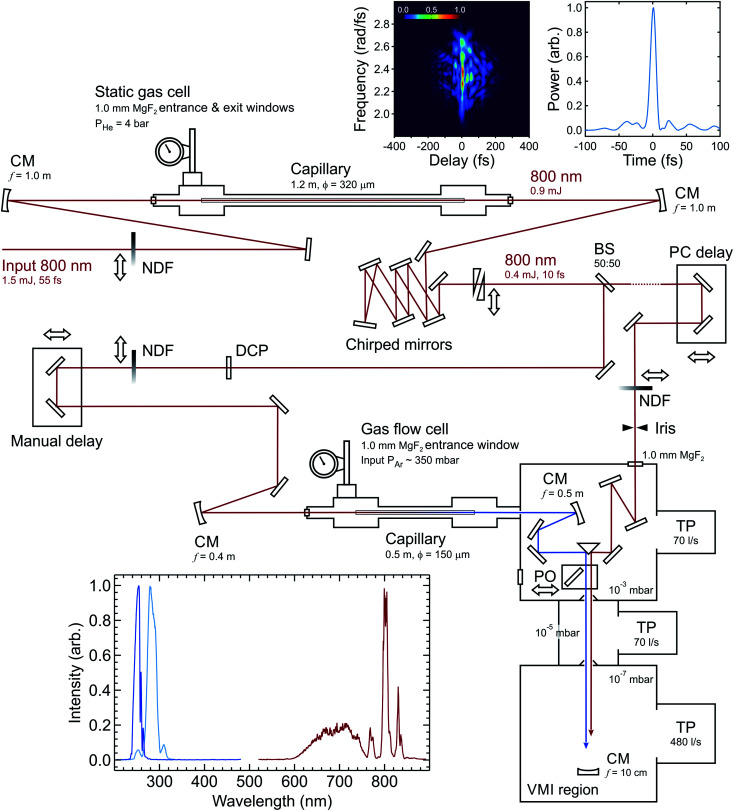
Schematic overview of the optical setup. Abbreviations: CM (concave mirror), BS (beamsplitter), DCP (dispersion compensator plate), NDF (neutral density filter), PO (pick-off optic), TP (turbomolecular pump). All energies quoted are per pulse. Cumulative reflection losses across the chirped mirror array are considerable due to deviation from near perpendicular incidence angles (as necessitated by space constraints). Top right inset panels show the second-harmonic generation frequency-resolved optical gating trace (left) and pulse profile (right) of the infrared beam after the chirped mirrors, with dispersion matched to the conditions in the interaction region. The pulse has a FWHM duration of 9.8 fs. Entrance and exit apertures on the middle differential pumping stage are *Ø*10 mm. For a more detailed description of the velocity-map imaging (VMI) region and overall spectrometer configuration, see ref. 39. The lower left inset panel shows (intensity normalized) traces of the broadband IR input spectrum that is coupled into the second (0.5 m) HCF, and the corresponding RDW output spectra centred at 250 nm and 280 nm. The intensity of the SPM broadened signal at wavelengths >850 nm is extremely weak due to a drop off in the response of the USB spectrometer (Ocean Optics, USB2000+) and fibre optic cable (Ocean Optics, QP400-2-UV-BX) used to make the measurement.

Fig. 1 shows a schematic of a novel experimental setup developed for TRPEI applications using an RDW source. Pulses from a 1 kHz Ti:sapphire laser system (800 nm central wavelength, 1.5 mJ pulse energy, 55 fs full width half maximum (FWHM) duration) are coupled into a first HCF (320 μm core diameter, 1.2 m length), which is filled with 4 bar of helium. This provides spectral broadening through self-phase modulation (SPM). The output pulses from this first HCF are then compressed to 10 fs duration by a set of six chirped mirrors arranged in a double-pass geometry (Thorlabs, UMC10-15FS), and a pair of translatable silica wedges for fine control of dispersion effects (Newport, 25RB12-01UF.AR2). A thin 50 : 50 broadband beamsplitter (Thorlabs, UFBS5050) is subsequently used to generate two independent optical pathways (each of ∼200 μJ per pulse) one of which provides the pump for our TRPEI measurements, and the other the probe. The latter – which is subsequently attenuated to ∼10 μJ per pulse using a neutral density filter – incorporates a retroreflector mounted on a motorized translation stage for accurate control of the temporal pump–probe delay.

**Fig. 2:**
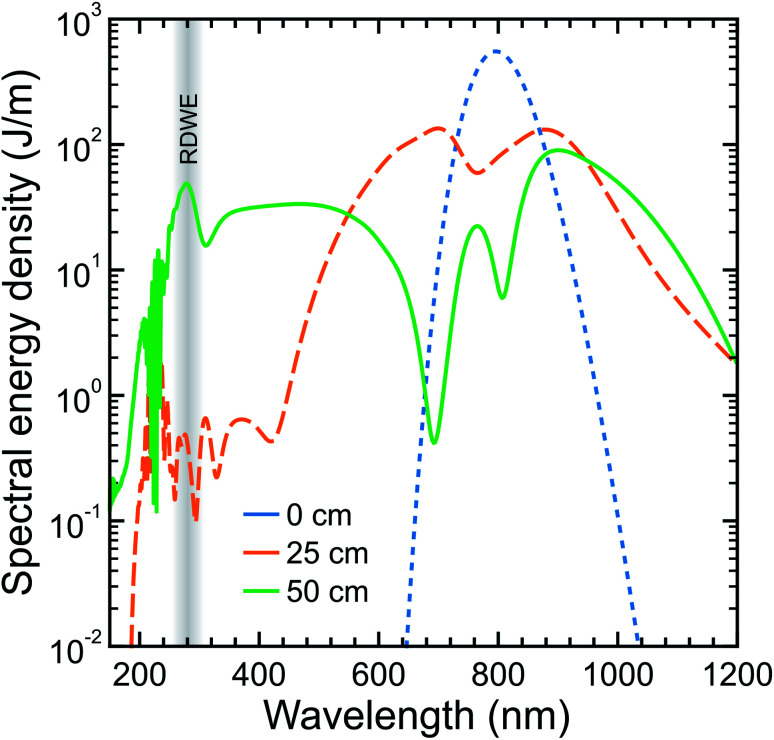
Numerical simulation of the optical spectrum evolution on propagating a 10 fs FWHM laser pulse centred at 800 nm through a 50 cm HCF with a 150 μm core diameter under decreasing gradient pressure for 500 mbar of argon. Blue, orange and green curves display cuts at the input, midway and output of the HCF, respectively. The region of resonant dispersive wave emission (RDWE) centred at 280 nm is highlighted. For more details on the pulse propagation model used in the simulation see ref. 33 and 40.

In the pump beamline, the 10 fs IR pulses are coupled into a second HCF (150 μm core diameter, 0.5 m length), which is directly connected to a vacuum system at the output end (described in more detail later). In this second HCF, the interplay between positive third-order nonlinearity and anomalous group-velocity dispersion leads to self-compression of the pulse and consequently the emission of a resonant dispersive wave. Fig. 2 shows a numerical simulation of this process at selected points along the HCF using parameters relevant to those in the current experiment. Initially, the propagating input pulse (blue dot trace in Fig. 2) experiences SPM due to the intensity-dependent refractive index of the HCF fill gas, leading to spectral broadening where the leading edge in time shifts to lower frequencies and the trailing edge shifts to higher frequencies (orange dash trace in Fig. 2). By adjusting the gas pressure such that the total (*i.e.* gas and waveguide) group velocity dispersion is anomalous at the input frequency, dispersion effects compensate for the non-linear chirp arising due to SPM. This then leads to pulse compression, which subsequently enhances further SPM and leads to a repeating cycle of continuous (soliton) self-compression. This process can extend the pulse spectrum by more than an octave, with higher-order dispersion then leading to a phase-matched (resonant) transfer of energy from the self-compressing pulse to a specific frequency in the DUV region (solid green trace in Fig. 2). The decreasing pressure gradient along the HCF removes the need for a dispersive exit window and is expected to lead to shorter DUV pulse duration at the HCF output.^36^ With 350 mbar of argon at the entrance of the HCF and vacuum (10^−3^ mbar) at the exit, we observe RDW emission centred at 250 nm with a FWHM bandwidth of ∼16 nm and an estimated pulse energy of ∼0.7 μJ. This estimate is based on a measurement at the point of interaction with a molecular sample (∼0.4 μJ per pulse) and then factoring in losses from the optics guiding the DUV beam from the HCF output to this point. Based on our ability to couple around 90 μJ per pulse into the second HCF, this corresponds to an efficiency of just under 1%. This can potentially be increased considerably by replacing the argon fill gas with neon or helium (as unwanted ionization effects are reduced).^33^ On the other hand, such a change necessitates the use of a far higher gradient pressure to generate the DUV output of interest and places much greater demands on the differential pumping requirements for coupling into a photoelectron spectrometer (as expanded upon below). In this initial set-up, a pulse energy of <0.5 μJ was deemed fully sufficient for our TRPEI applications.

The 250 nm DUV output spectrum from the second HCF is shown in the inset panel of Fig. 1 along with that of the initial IR input into this HCF. Based on this data, the Fourier-transform-limited duration of the DUV pulse is expected to be around 5–6 fs. By adjusting the argon pressure between 300 and 600 mbar, the central wavelength of the DUV output can be varied easily between 240 and 300 nm (an example at 280 nm is also included in Fig. 1). As has been demonstrated elsewhere, however, a far greater tuning range is possible in HCF RDW emission^34^ and the spectral limits are only restricted in the current setup by the fill gas and broadband coatings on the mirrors used to manipulate the DUV beam after generation. The DUV output is currently of sufficient stability to permit spectroscopic measurements over periods of approx. 4 hours – which is adequate for many applications. Small fluctuations in laser pointing lead, however, to the extremely high intensity of the input 800 nm degrading the capillary tip and reducing efficiency in the longer term. The use of an active beam stabilization system is expected to significantly improve this situation (based on our experiences with other RDW setups we have developed) and this will be implemented in a future experimental upgrade. In the meantime, day-to-day performance is reproducible upon replacing a damaged capillary with a new section.

## Initial characterization

We characterize our experimental instrument response function using non-resonant two-colour DUV-IR pump–probe ionization of 1,3-butadiene in a photoelectron spectrometer optimized for velocity map imaging (VMI).^41^ A detailed description of the main instrument has been given elsewhere.^39^ In brief, this spectrometer consists of two independently pumped ultra-high vacuum chambers arranged vertically and connected *via* a small orifice skimmer (*Ø*1 mm). The lower chamber is used for the preparation of the (undiluted) 1,3-butadiene molecular beam, which is generated by continuous effusive expansion through a pinhole nozzle (*Ø*150 μm). This sits below the plane of optical propagation and is omitted from Fig. 1. The upper chamber houses an electrostatic lens assembly where the interaction between the molecular sample beam and the laser pulses takes place. An additional vacuum chamber connected to the upper spectrometer chamber is used for spectral filtering and steering of the DUV pulses. After leaving the HCF, the beam is collimated in this additional chamber by an aluminium focusing mirror. A pair of broadband dichroic mirrors (Edmund Optics 47-985 or Layertec 110132) then separate the DUV pulses from the co-propagating residual IR and visible supercontinuum. The dichroic and aluminium mirrors are mounted on motorized optical mounts for in-vacuum beam steering. Once propagating under vacuum, the pump and probe beams are guided towards the two perpendicular faces of an aluminium-coated right-angle prism and then reflected into the main photoelectron spectrometer in a parallel, copropagating geometry. A small auxiliary chamber serves as a differential pumping stage between this region and the main spectrometer, keeping the pressure in the VMI region low (10^−7^ mbar) despite relatively high pressure at the exit of the HCF (10^−3^ mbar). Alternatively, a pick-off mirror mounted on a small, motorized translation stage can be inserted into the optical path to divert the beams out of the starting vacuum chamber for preliminary beam alignment, spectroscopic monitoring, and power measurements. The requirement for small apertures between the various differential pumping stages means that the diameter of the pump and probe beams are set at ∼2 mm. This is achieved by the choice of curved mirrors for beam recollimation after each capillary stage and (in the case of the 800 nm probe) an iris. Small beams are also helpful as they limit the peak intensity at the focused interaction point with a molecular sample.

**Fig. 3:**
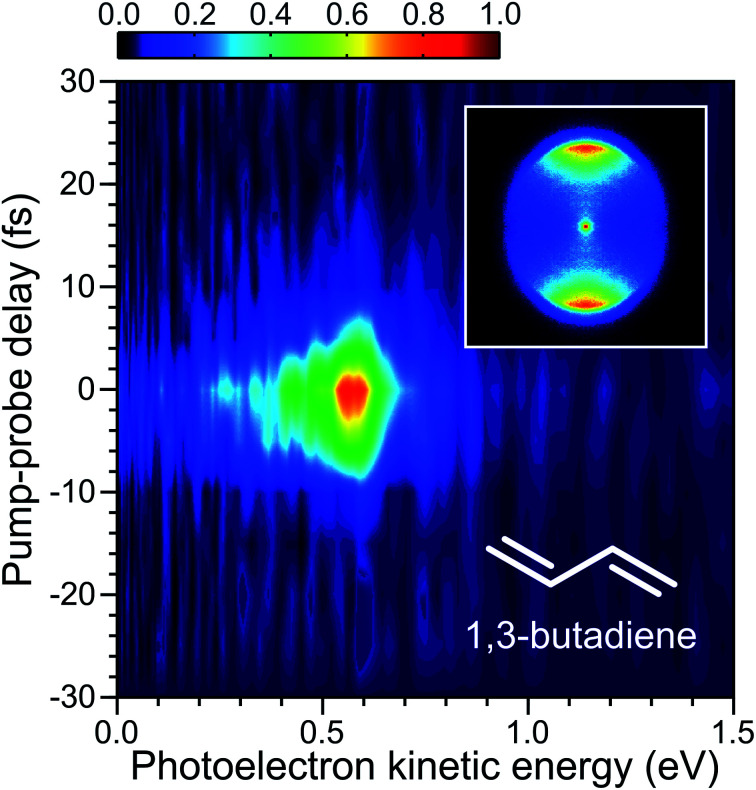
Time-dependent photoelectron spectrum of 1,3-butadiene obtained using a 250 + 800 nm, 1 + 3′ ionization scheme. A photoelectron image obtained close to zero pump–probe delay is inset. This image has been 4-fold symmetrized (*i.e.* the raw data in four individual quadrants defined by horizontal and vertical axes passing though the image centre have been averaged together – which is valid given the expected symmetry of the photoelectron angular distribution). The pump and probe polarizations are vertical with respect to the inset figure.

Upon entering the main vacuum chamber of the spectrometer, the unfocused DUV pump and IR probe beams initially pass straight through the electrostatic VMI lenses before reflecting off a curved aluminium mirror (*f* = 10 cm) attached to a high-precision *x*–*y*–*z* manipulator. Ionization of the molecular sample by the tightly focused beams then occurs on a second optical pass back through the VMI region. The photoelectrons produced are accelerated along a short (26 cm) flight tube and then imaged using a 40 mm diameter dual micro-channel plate/P47 phosphor screen detector in conjunction with a CCD camera (640 × 480 pixels). The inset in Fig. 3 shows an example VMI image. We use a fast matrix inversion method for image processing to provide data in a form suitable for subsequent angle-, time- and energy-resolved analysis. With appropriate pixel-to-energy calibration, this permits the extraction of time-resolved photoelectron spectra. Expanded details may be found elsewhere.^39^

For the initial TRPEI measurements using 1,3-butadiene, ionization occurs *via* the absorption of one DUV photon and three IR photons (which we label as a 1 + 3′ event). At the DUV wavelengths considered here, this process is non-resonant and leads to a simple cross-correlation measurement that defines the instrument response function (and therefore the limit of any temporal resolution). A time-resolved photoelectron spectrum is shown in Fig. 3 for the case where the RDW emission in the DUV is centred at 250 nm. This spectrally resolved cross-correlation data exhibits no evidence of any temporal shift in the position of maximum signal intensity as a function of photoelectron energy. This implies that the pump and probe pulses are not significantly chirped, and dispersion effects are adequately controlled – something that is only possible with the extremely broadband DUV pulses due to propagation under vacuum conditions after exiting the HCF.

**Fig. 4:**
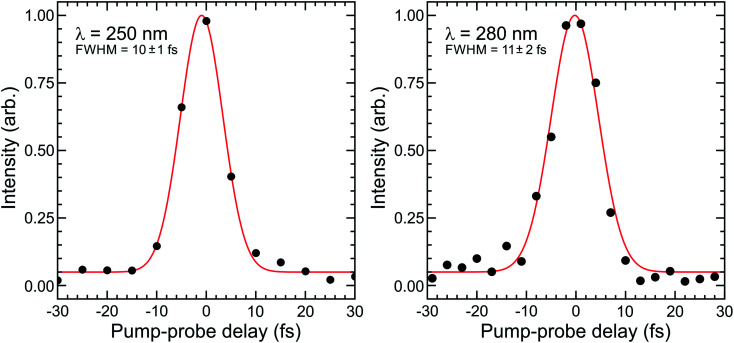
Energy integrated photoelectron transients obtained from 1 + 3′ non-resonant DUV-IR photoionization of 1,3-butadiene at DUV pump wavelengths centred at 250 and 280 nm. Gaussian fits to these data (solid red lines) are also overlaid (quoted uncertainties are 1*σ* values).

Fig. 4 shows Gaussian fits to energy integrated TRPEI data recorded at DUV wavelengths centred at 250 and 280 nm. The FWHM obtained in the fits are 10 ± 1 and 11 ± 2 fs, respectively. This is extremely short and represents an advance in the current state of the art for TRPEI measurements.^42–44^ Given the ionization is a 1 + 3′ process overall and considering the individual IR probe pulse duration of 10 fs (measured *via* second-harmonic generation frequency-resolved optical gating – see Fig. 1 top inset), this corresponds to a DUV pulse of ∼6 fs. This is in good agreement with the transform limited prediction based on the optical RDW output spectrum. Focused intensities are of order 10^13^ W cm^−2^. This is relatively high and illustrates that pulse energies <0.5 μJ are easily sufficient for many pump–probe spectroscopic applications exploiting the extremely short temporal durations of RDW sources. It also provides a caveat that any future scaling up of the output energy/efficiency should be used with a degree of caution, particularly when it is desirable to maintain a regime where molecular dynamics are passively observed rather than actively modified. For the present example of 1,3-butadiene – as well as that of *N*-methylpyrrolidine discussed later – Keldysh parameters are >1, indicating that strong field tunnel ionization effects are not expected to contribute to our observed photoelectron signals.^45^ This is a relatively limited approximation for molecular ionization,^46^ but still serves as a useful indicative benchmark when evaluating experimental conditions. Furthermore, field-induced Stark shifts may, for example, exert a degree of control over photoproduct branching ratios.^47,48^ The strength of such (polarizability driven) effects is highly molecule/electronic state dependent, but is not expected to be a significant factor in the work presented here. This is based on variable intensity ionization measurements conducted on structurally related molecular systems that will form the basis of a future publication.^49^

The findings presented in Fig. 3 and 4 provide a powerful demonstration of the potential for RDW-based optical sources to significantly enhance temporal resolution in ultrafast spectroscopy over an easily tuneable range of DUV wavelengths. While the data presented here are of direct relevance to TRPEI measurements – as further demonstrated below – we anticipate our approach will also be suitable for use in many other time-resolved applications, leading to much wider impact within the ultrafast spectroscopy community.

## Application to dynamics measurements

Having successfully characterized our experimental setup, we present an initial TRPEI study investigating excited state dynamics in the saturated tertiary amine *N*-methylpyrrolidine. The amine functional group is a commonly occurring motif in organic chemistry and in nature, appearing, for example, in the DNA bases, amino acids and neurotransmitters. This leads to an interest in determining how the structure surrounding the nitrogen chromophore influences the overall system photodynamics following ultraviolet absorption.^21,50–58^ Furthermore, the predominantly Rydberg character of the exited states leads to a strong propensity for Δ*v* = 0 ionizing transitions to the cation (where *v* is a generalized, non-mode-specific vibrational quantum number), giving rise to well resolved photoelectron bands. This, along with their relatively low ionization potentials (typically <8.5 eV) and high vapor pressures, makes amines excellent candidate species for an initial demonstration of our RDW source. The experimental setup is identical to that described earlier, although liquid samples of *N*-methylpyrrolidine are now retained in a bubbler vessel external to the vacuum system and helium (0.5 bar) is used as a carrier gas for the effusive molecular beam.

**Fig. 5:**
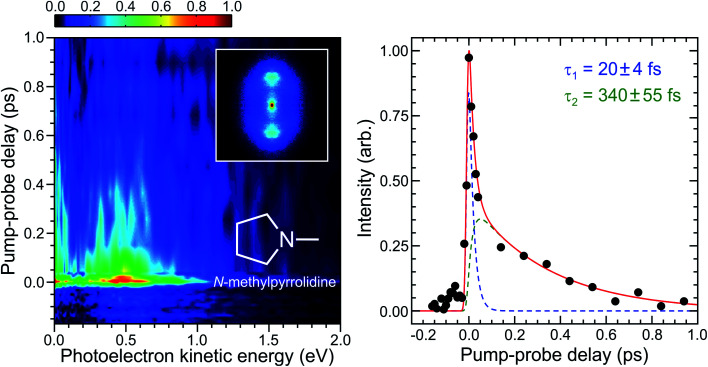
(Left) Time-dependent photoelectron spectrum of *N*-methylpyrrolidine obtained using a 250 + 800 nm, 1 + 3′ ionization scheme. Time invariant pump-alone and probe-alone signals have been background subtracted. A photoelectron image obtained close to zero pump–probe delay is inset. As in Fig. 3, this has been 4-fold symmetrized and the pump/probe polarizations are vertical. (Right) Corresponding energy integrated photoelectron transient (0.1–1.0 eV range) and fit to this data using a sequential bi-exponential model described in the main text (solid red line). Individual fit components are also included (blue and green dashed lines). Quoted uncertainties in numerical time constants are 1*σ* values.

A time-dependent photoelectron spectrum of *N*-methylpyrrolidine obtained using a 250 + 800 nm pump–probe ionization scheme is presented in the left-hand panel of Fig. 5. There is a very rapidly evolving transient signal (<50 fs) evident in this data, as well as a longer-lived decay on the order of a few hundred femtoseconds. A weak “probe–pump” signal can also be seen extending in the direction of negative delay time. As was the case with 1,3-butadiene, the overall pump–probe scheme is 1 + 3′, although now the pump can resonantly excite the 3s state of *N*-methylpyrrolidine. The ultraviolet absorption spectrum has been presented in our previous work on this molecule, with the 3s state giving rise to a broad, structureless band that has an onset close to 260 nm.^21^

The temporal evolution in our TRPEI data is further illustrated in the right-hand panel of Fig. 5, which plots the overall transient signal (integrated over the first eV of photoelectron kinetic energy) as well as a fit to the data using an expression of the following form:1
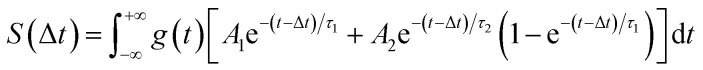
Here Δ*t* is the pump–probe delay time, *g*(*t*) denotes the experimentally determined Gaussian cross-correlation function (10 fs FWHM, as determined in Initial characterization), with *A*_i_ and *τ*_i_ being the amplitude and 1/*e* lifetime of a given fit function, respectively. This simple, two-step sequential model yields fit lifetimes of *τ*_1_ = 20 ± 4 and *τ*_2_ = 340 ± 55 fs. Detailed evaluation and discussion of the data presented in Fig. 5 is beyond the scope of this initial TRPEI measurement, and an expanded study, including full analysis of the photoelectron angular distributions, will form the basis of a future publication (where a considerable degree of additional theoretical input will also be required). Provisionally, however, we assign *τ*_1_ to the rapid dephasing of an initially localized vibrational wavepacket on the 3s state potential energy surface – a feature that would have been challenging to definitively resolve without the excellent temporal resolution made available by our new set-up. Here, however, such extremely rapid dynamics is unambiguously observed. Within the 1 ps observation window of our experiment we see no clear evidence of any coherent oscillations that would indicate the revival of such a wavepacket, although this may be obscured given the limited number of pump–probe delay points sampled beyond 100 fs in this preliminary measurement. The more striking result here is the sub-picosecond dynamics described by *τ*_2_, which we attribute to the overall lifetime of the 3s state. Earlier TRPEI studies conducted on *N*-methylpyrrolidine using a 200 nm pump have optically prepared the 3p Rydberg manifold and observed rapid 3p → 3s internal conversion in less than 1 ps.^21^ The 3s state was then observed to exhibit an extended lifetime of 160 ps. Given the far greater levels of resultant 3s vibrational excitation in this 200 nm pump scenario, the much shorter 340 fs lifetime now seen following the lower-energy excitation at 250 nm is an unexpected outcome. This is particularly true given a study of the related *N*,*N*-dimethylisopropylamine system has revealed lifetimes extending to over a nanosecond towards the red end of the 3s absorption band.^59^ We note, however, that quantum yield studies in several other tertiary amine systems suggest fluorescence is an important decay pathway at excitation wavelengths >220 nm,^60–63^ while nonradiative energy redistribution pathways become predominant following higher energy absorption. Of particular relevance here is a fluorescence lifetime study reporting dual exponential decay in several amine systems following measurements that excite the 3s state and 3p manifold simultaneously.^60^ This was rationalized by arguing different vibrational distributions are produced in the 3s state following direct optical preparation and when populated indirectly *via* internal conversion. Such differences then lead to the latter pathway having a reduced propensity for fluorescence decay. Related ideas have also been put forward based on more recent results from time-resolved mass spectrometry measurements investigating deuteration effects in various amines. Here parent ion lifetimes were observed to increase for more massive N atom substituents.^54^ This is counterintuitive to heuristic “density of states” arguments and was interpreted as a signature of non-ergodic phenomena (*i.e.* when the nuclear dynamics only sample a reduced phase space prior to internal conversion).^64^ Based on these observations, we provisionally suggest some form of related effect may be in operation here, with the population of different subsets of vibrational modes leading to a shorter 3s state lifetime in *N*-methylpyrrolidine following 250 nm excitation when compared to 200 nm. A more expansive wavelength-dependent study of this process will reveal much more detail in this regard, but we note a similar behaviour reported previously in the styrene molecule.^65^ Importantly, however, our initial findings prove the suitability of the vacuum integrated RDW source for practical spectroscopic applications, which is a key demonstration if the general approach is to be adopted more widely.

## Conclusions

We have successfully demonstrated the generation of tuneable few-femtosecond deep ultraviolet pulses for ultrafast spectroscopy applications using RDW emission in hollow capillary fibres. Integrating the output end of a such a fibre directly into a vacuum chamber assembly means optical dispersion effects are effectively eliminated, permitting two-colour DUV-IR instrument response functions as short at 10 fs to be measured using the TRPEI technique. We have also presented a first dynamics measurement with this vacuum integrated RDW source, conducting an initial TPPEI study of *N*-methylpyrrolidine using a 250 nm pump. This unambiguously reveals some rapid (20 fs) temporal evolution that would be challenging to resolve definitively using more conventional UV generation strategies. Intriguing new insight about the potential for wavelength-dependent competition between different decay pathways in amine systems is also suggested, and this will form the basis of more extended future investigations. More generally, the extremely short pulses and relative ease of broad DUV (and potentially VUV) tunability afforded by RDW sources offers enormous potential to transform a wide range of ultrafast spectroscopy applications over the next decade and beyond. We expect that our initial validation of this approach will generate important stimulus in this area, motivating uptake of this exciting new technology. Through refinements to our optical setup, we anticipate extending the RDW bandwidth in the DUV to more than 20 nm, reducing the concomitant pulse duration to <5 fs. Future upgrades to vacuum pumps will also permit integration of a second capillary into the system, permitting fully tuneable DUV + DUV (or VUV) pump–probe measurements. Such upgrades are required due to the increased gas handling requirements associated with the higher pressures produced from two differentially pumped fibres.

## Data availability

The data that support the findings of this study are available from the corresponding author upon reasonable request.

## Author contributions

D. Townsend and J. C. Travers supervised the overall project. N. Kotsina and C. Brahms designed and built the RDW optical beamline. N. Kotsina and S. L. Jackson undertook the TRPEI measurements. N. Kotsina analysed the data. D. Townsend wrote the paper (with contributions from N. Kotsina and C. Brahms). All authors contributed to the discussion of the results.

## Conflicts of interest

There are no conflicts to declare.

## Supplementary Material

## References

[cit1] Mustroph H. (2016). ChemPhysChem.

[cit2] Schuurman M. S., Stolow A. (2018). Annu. Rev. Phys. Chem..

[cit3] Mahapatra S. (2008). Acc. Chem. Res..

[cit4] Worth G. A., Cederbaum L. S. (2004). Annu. Rev. Phys. Chem..

[cit5] Yarkony D. R. (2001). J. Phys. Chem. A.

[cit6] Baker L. A., Grosvenor L. C., Ashfold M. N. R., Stavros V. G. (2016). Chem. Phys. Lett..

[cit7] Maeda S., Taketsugu T., Ohno K., Morokuma K. (2015). J. Am. Chem. Soc..

[cit8] Pollum M., Crespo-Hernández C. E. (2014). J. Chem. Phys..

[cit9] Cui G., Fang W. (2013). J. Chem. Phys..

[cit10] Tully J. C. (2012). J. Chem. Phys..

[cit11] Pederzoli M., Pittner J., Barbatti M., Lischka H. (2011). J. Phys. Chem. A.

[cit12] Sundström V. (2008). Annu. Rev. Phys. Chem..

[cit13] Kukura P., McCamant D. W., Yoon S., Wandschneider D. B., Mathies R. A. (2005). Science.

[cit14] Crespo-Hernández C. E., Cohen B., Hare P. M., Kohler B. (2004). Chem. Res..

[cit15] Kotsina N., Townsend D. (2021). Phys. Chem. Chem. Phys..

[cit16] Wu G., Hockett P., Stolow A. (2011). Phys. Chem. Chem. Phys..

[cit17] Suzuki T., Whitaker B. J. (2001). Int. Rev. Phys. Chem..

[cit18] Paterson M. J., Townsend D. (2020). Int. Rev. Phys. Chem..

[cit19] Svoboda V., Bhargava Ram N., Rajeev R., Wörner H. J. (2017). J. Chem. Phys..

[cit20] Zawadzki M. M., Candelaresi M., Saalbach L., Crane S. W., Paterson M. J., Townsend D. (2016). Faraday Discuss..

[cit21] Thompson J. O. F., Klein L. B., Sølling T. I., Paterson M. J., Townsend D. (2016). Chem. Sci..

[cit22] Horke D. A., Watts H. M., Smith A. D., Jager E., Springate E., Alexander O., Cacho C., Chapman R. T., Minns R. S. (2016). Phys. Rev. Lett..

[cit23] Horio T., Spesyvtsev R., Nagashima K., Ingle R. A., Suzuki Y.-I., Suzuki T. (2016). J. Chem. Phys..

[cit24] Thompson J. O. F., Saalbach L., Crane S. W., Paterson M. J., Townsend D. (2015). J. Chem. Phys..

[cit25] Suzuki Y.-I., Horio T., Fuji T., Suzuki T. (2011). J. Chem. Phys..

[cit26] Bisgaard C. Z., Clarkin O. J., Wu G., Lee A. M. D., Gessner O., Hayden C. C., Stolow A. (2009). Science.

[cit27] Reiter F., Graf U., Schultze M., Schweinberger W., Schröder H., Karpowicz N., Azzeer A. M., Kienberger R., Krausz F., Goulielmakis E. (2010). Opt. Lett..

[cit28] Galli M., Wanie V., Lopes D. P., Månsson E. P., Trabattoni A., Colaizzi L., Saraswathula K., Cartella A., Frassetto F., Poletto L., Légaré F., Stagira S., Nisoli M., Vázquez R. M., Osellame R., Calegari F. (2019). Opt. Lett..

[cit29] Baum P., Lochbrunner S., Riedle E. (2004). Opt. Lett..

[cit30] Bruder L., Wittenbecher L., Kolesnichenko P. V., Zigmantas D. (2021). Opt. Express.

[cit31] Joly N. Y., Nold J., Chang W., Hölzer P., Nazarkin A., Wong G. K. L., Biancalana F., Russell P. S. J. (2011). Phys. Rev. Lett..

[cit32] Mak K. F., Travers J. C., Hölzer P., Joly N. Y., Russell P. S. J. (2013). Opt. Express.

[cit33] Travers J. C., Grigorova T. F., Brahms C., Belli F. (2019). Nat. Photonics.

[cit34] Brahms C., Belli F., Travers J. C. (2020). Phys. Rev. Res..

[cit35] Brahms C., Austin D. R., Tani F., Johnson A. S., Garratt D., Travers J. C., Tisch J. W. G., Russell P. S. J., Marangos J. P. (2019). Opt. Lett..

[cit36] Brahms C., Belli F., Travers J. C. (2020). Opt. Lett..

[cit37] Brahms C., Travers J. C. (2021). J. Phys.: Photonics.

[cit38] Kotsina N., Belli F., Gao S., Wang Y., Wang P., Travers J. C., Townsend D. (2019). J. Phys. Chem. Lett..

[cit39] Livingstone R. A., Thompson J. O. F., Iljina M., Donaldson R. J., Sussman B. J., Paterson M. J., Townsend D. (2012). J. Chem. Phys..

[cit40] https://github.com/LupoLab/Luna.jl, 10.5281/zenodo.5513570

[cit41] Eppink A. T. J. B., Parker D. H. (1997). Rev. Sci. Instrum..

[cit42] Suzuki Y.-I., Fuji T., Horio T., Suzuki T. (2010). J. Chem. Phys..

[cit43] Horio T., Spesyvtsev R., Suzuki T. (2013). Opt. Express.

[cit44] Kobayashi T., Horio T., Suzuki T. (2015). J. Phys. Chem. A.

[cit45] Keldysh L. V. (1965). Sov. Phys. JETP.

[cit46] DeWitt M. J., Levis R. J. (1998). Phys. Rev. Lett..

[cit47] Townsend D., Sussman B. J., Stolow A. (2011). J. Phys. Chem. A.

[cit48] Sussman B. J., Townsend D., Ivanov M. Y., Stolow A. (2006). Science.

[cit49] Kotsina N. , Jackson S. L., Malcomson T., Paterson M. J. and Townsend D., in preparation 10.1039/d2cp04789f 36453640

[cit50] Du W., Gao Y., Stankus B., Xu X., Yong H., Weber P. M. (2021). Phys. Chem. Chem. Phys..

[cit51] Waters M. D. J., Skov A. B., Larsen M. A. B., Clausen C. M., Weber P. M., Sølling T. I. (2019). Phys. Chem. Chem. Phys..

[cit52] Klein L. B., Thompson J. O. F., Crane S. W., Saalbach L., Sølling T. I., Paterson M. J., Townsend D. (2016). Phys. Chem. Chem. Phys..

[cit53] Klein L. B., Morsing T. J., Livingstone R. A., Townsend D., Sølling T. I. (2016). Phys. Chem. Chem. Phys..

[cit54] Klein L. B., Sølling T. I. (2014). Chem. Phys..

[cit55] Deb S., Cheng X., Weber P. M. (2013). J. Phys. Chem. Lett..

[cit56] Deb S., Minitti M. P., Weber P. M. (2011). J. Chem. Phys..

[cit57] Deb S., Bayes B. A., Minitti M. P., Weber P. M. (2011). J. Phys. Chem. A.

[cit58] Bush J. C., Minitti M. P., Weber P. M. (2010). J. Phys. Chem. A.

[cit59] Rudakov F., Zhang Y., Cheng X., Weber P. M. (2013). Opt. Lett..

[cit60] Cureton C. G., Hara K., O'Connor D. V., Phillips D. (1981). Chem. Phys..

[cit61] Matsumi Y., Obi K. (1980). Chem. Phys..

[cit62] Abbott G. D., Cureton C. G., Hara K., Hirayama S., Phillips D. (1978). J. Photochem..

[cit63] Halpern A. M. (1974). J. Am. Chem. Soc..

[cit64] Sølling T. I., Kuhlman T. S., Stephansen A. B., Klein L. B., Møller K. B. (2014). ChemPhysChem.

[cit65] Nunn A. D. G., Minns R. S., Spesyvtsev R., Bearpark M. J., Robb M. A., Fielding H. H. (2010). Phys. Chem. Chem. Phys..

